# The benefit of cinnamon ( *Cinnamomum burmannii*) in lowering total cholesterol levels after consumption of high-fat containing foods in white mice ( *Mus musculus*) models

**DOI:** 10.12688/f1000research.22311.2

**Published:** 2020-05-29

**Authors:** Annisa Pulungan, Yunita Sari Pane

**Affiliations:** 1 Faculty of Medicine, Universitas Sumatera Utara, Medan, North Sumatera, Indonesia; 2 Department of Pharmacology, Faculty of Medicine, Universitas Sumatera Utara, Medan, North Sumatera, Indonesia

**Keywords:** hypercholesterolemia, cinnamon, high-fat feed, quail yolk, body weight of mice

## Abstract

**Background:** Hypercholesterolemia is a condition where cholesterol levels in the body exceed the normal range. If the condition is longer, it can cause metabolic and cardiovascular diseases. The therapy of synthetic drugs has side effects that can be fatal (rhabdomyolysis). Needed to find natural remedies with minimal side effects. There are many nutritional components contained in cinnamon, such as cinnamaldehyde. The cinnamaldehyde, a substance that is thought to affect cholesterol levels. The study aims to determine the efficacy of *Cinnamomum burmannii* in lowering total cholesterol levels of mice *(Mus musculus*) given high-fat feed.

**Methods: **This is an experimental study with a pre-post control study design. The groupings were performed by a simple random sampling method. The male mice were divided into five groups (n=6/group): 1) negative control (aquadest); 2) positive control of high-fat containing food (HFC; quail yolk); 3) HFC + cinnamon extract (CE; dose 2mg/20g body weight (BW); 4) HFC + CE (dose 4mg/20gBW); 5) HFC + CE (dose 8mg/20gBW). The study was conducted for 28 days. Consumption of quail yolk as HFC to increasing cholesterol in mice. The intervention of CE started on day 15 and ended on day 28. Measurement of total cholesterol and BW of mice was performed on days 0, 14 and 28.

**Results: **The comparison of total cholesterol levels in the K1 group (120.3 ± 5.53 mg/dl) to K2 (107.3 ± 3.61 mg/dl), K3 (106.8 ± 4.57 mg/dl) and K4

(106.7 ± 0.51 mg/dl) showed decreased significantly different (p = 0.001) in groups consuming CE. However, there was not a significant change between groups in mice BW (p = 0.419).

**Conclusions**: The cinnamon ( *Cinnamomum burmannii*) proved can be lowering of total cholesterol levels for 14 days in mice compared without given cinnamon after consumption of high-fat containing foods.

## Introduction

Hypercholesterolemia is a state where the cholesterol level in the body exceeds the normal range. Hypercholesterolemia can increase the risk of atherosclerosis, coronary artery disease, pancreatitis, diabetes mellitus, liver disease and renal disease, etc ^1^.

Data from the Indonesian Ministry of Health in 2019 reported that coronary artery disease to be the first killer, 26.4% of all deaths in Indonesia ^2^. Out of the 17 million the premature death (under the age of 70) due to non communicable disease, most cases are in low-and middle income countries, and 37% are caused coronary heart disease and 6.7 million of these were triggered by stroke (WHO, 2015) ^3^.

Many side effects caused by the condition of hypercholesterolemia need good care besides improving lifestyle by reducing the intake of high fat foods, also taking drugs, such as statin drugs. Ezad *et al.* (2018) reported that the use of statin drugs can cause side effects such as myopathy, increase levels of liver enzymes, increase the risk of impairment or memory loss, and can even be fatal caused rhabdomyolysis. Therefore, it is important to find natural remedies that are efficacious in reducing cholesterol levels with minimal side effects ^4^. In Indonesia, the development of traditional medicine is performed by exploring herbs known to be beneficial for health, and this information is passed from generation to generation. One such herb is *gambier (Uncaria gambir* Roxb. *)* ^5^, which has antioxidant effects and lowers blood glucose when treating type 2 diabetes mellitus (T2DM). There are also *bangun-bangun* leaves ( *Coleus amboinicus*), which are believed to be effective in relieving pain when distilled as ethanol and water extract ^6^. Traditional medicine that comes from plants is commonly used by many people in Indonesia. One of these herbs is cinnamon ( *Cinnamomum burmannii*). It has the efficacy to lower blood glucose levels. It is potential as an antidiabetic because it contains high levels of cinnamaldehyde. Cinnamon is often eaten in daily food. Cinnamon is a native plant in Indonesia and can be found abundant in Central of Java, (Karanganyar), West Sumatra (Padang), Jambi (Kerinci), etc ^7^.

The consuming high fat feed can increase cholesterol in the blood. Lipid levels of yolks of quail are higher than chicken egg yolks ^8^. Preliminary studies have shown success in creating hypercholesterolemia mice models by consuming high-fat feed using quail yolk. Mice were used in this study, because they have biology similar to humans, and therefore can be a model for human hyperlipidemia ^9– 11^. This study was conducted to determine the efficacy of *Cinnamon burmannii* in lowering total cholesterol levels of mice ( *Mus musculus*) given high-fat feed.

## Methods

This is an experimental study in an animal model with a pre-post control study design. This study was conducted at the Pharmacology Laboratory of the Faculty of Medicine, Universitas Sumatera Utara, Indonesia.

### Ethics

This study was approved by the Health Research Ethical Committee of Universitas Sumatera Utara (No:55/TGL/KEPK FK USU-RSUP HAM/2019).

Mice were given high-fat containing food (HFC) to create hyperlipidemic mouse model. The intervention was carried out by giving different CE doses to find the dose that reduces the total cholesterol level of the mice. Cholesterol total levels were obtained from the blood by cutting the mice’s tail. To ensure the experimental animals remained in a comfortable condition, the mice were given anesthesia procedures before cutting off the tail ^12, 13^.

### Experimental animals

The animals were purchased from the Department Biology of Mathematics and Scientific Faculty USU. In total, 30 male white mice ( *Mus musculus*), Swiss Webster strain, 10 to 12 weeks old, and weighing 25 – 40 g were used. Before conducting the study, the mice were adapted to their cages (plastic (30 × 20 × 10 cm) and covered with fine wire mesh; base of the cages was covered with rice husks as thick as 0.5 – 1 cm and replaced every day during the study) for 2 weeks before the experiment started. They had 12 hours of daylight (6:00 A.M. – 6:00 P.M.) and 12 hours of dark (6:00 P.M. – 6:00 A.M.). The mice were fed with standard feed (CP 551) from PT Charoen Pokphand-Indonesia and water was given *ad libitum*. Room temperature and humidity were kept at normal ranges. They were weighed once a week to avoid stress.

### Allocation and treatment groups

After the adaptation period, the mice were randomly divided into five groups, with each group consisting of 6 mice.

The sample size was calculated according to Federer’s formula ^14^:

(t-1) (n-1)>15

t = the number of groups

n=the number of samples

All 30 mice were given a number (1–30) using a SPIDOL marker pen, then randomized by putting the numbers in an envelope and dividing them into 5 groups according to the numbers are taken from the envelope ^9^.

Groups were as follows: 1) K0, negative control group/placebo, no treatment or high-fat food (only given aquadest 0.5 ml); 2) K1, positive control group, diet of HFC (quail yolk); 3) K2, HFC + cinnamon extract (CE) dose 2mg/20g body weight (BW); 4) K3, HFC + CE dose 4mg/20gBW; 5) K4, HFC + CE dose 8mg/20gBW.

The study was conducted for 28 days.

***Cinnamon extract.*** CE was given to the mice every morning at 8:00 am. Animals were kept during research in the mice laboratory in the Pharmacology laboratory. Before being given CE, it was dissolved with aquadest. The extract was given to the mice orally using feeding tubes. CE interventions began on the 15th day until the 28th day.

*Cinnamon extract* ( *Cinnamomum burmannii*) was obtained as Herbilogy Cinnamon Extract Powder® (PT.Phytochemindo Reksa, Bogor, Indonesia; batch no. 033CT).

***High-fat containing food.*** The quail yolk was used to induce hypercholesterolemia, because the concentration of the lipid in quail yolk is higher than chicken egg yolk ^8^. A dosage of 0.5ml/day was given until day 28. The administration of quail yolk was done orally using feeding tubes as per the administration of CE.

***Determination of dose of cinnamon.*** Based on research by Vanessa *et al.* in 2013, who saw a decrease in total blood cholesterol levels in white rats ( *Rattus norvegicus*) by administering instant cinnamon powder drink (C *innamomum burmannii* BI.) at a dose of 14.4 mg and 43.2 mg for 7 days. In their research, it was found that a dose of 14.4 mg can reduce cholesterol levels in rats. Therefore we used a dose of 14.4 mg, because it was effective and efficient ^15^. We converted its dose to mice and increased it with variation doses of 2, 4 and 8 mg. The value converted from 200g rat to 20g mice is 0.14.

Ratdose=14.4mg

Conversiontomice=0.14×14.4mg=2.01mg

~2mg/20gBWmice

### Laboratory analysis

Measurement of total cholesterol and BW of mice was performed on days 0, 14 and 28 (every two weeks). Anesthesia ketamine/xylazine 0.1 ml was injected intraperitoneal before the mice’s tail cutting off ^12, 13^.

Mice tails were cut 2 – 5mm to draw blood, which was then assessed for total cholesterol using the digital *autocheck ^®^* cholesterol measurement tool. Mice were weighed using a digital scale.

### Statistical analysis

Data were analyzed using SPSS 24. The number average of sample data presented as mean ± SD. The one-way ANOVA statistical analysis to indicate the effects of treatments for all group. If the result is significant (p < 0.05), then bootstrapping was performed using *post-hoc* Bonferroni for differentiating between each group.

## Results

**Figure 1.**
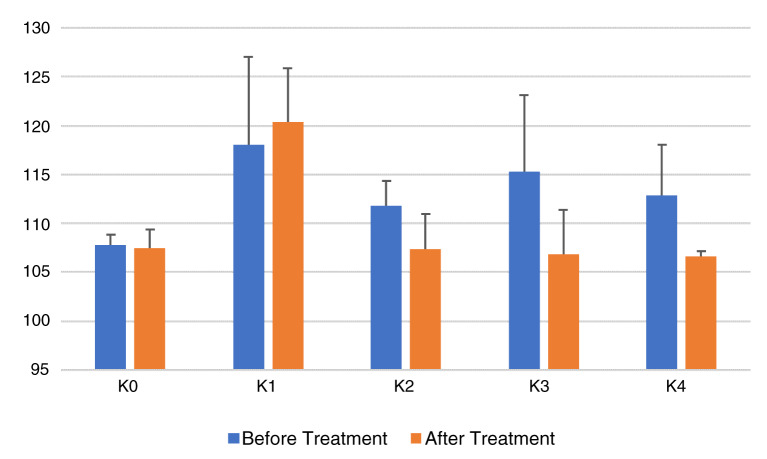
Total cholesterol per treatment group before (day 14) and after (day 28) treatment. K0, negative control group, no treatment or high-fat food (aquadest); K1, positive control group, diet of high-fat containing food (HFC; quail yolk); K2, HFC + cinnamon extract (CE) dose 2mg/20g body weight (BW); K3, HFC + CE dose 4mg/20gBW; K4, HFC + CE dose 8mg/20gBW.

Figure 1 shows that groups K0 (negative controls), K1 (positive controls), K2 (CE 2 mg/20gBW), K3 (CE 4 mg/20gBW), and K4 (CE 8 mg/20gBW), had a decrease in total cholesterol levels. There is a significant difference in total cholesterol among the groups (p=0.001 between groups). A post-hoc Bonferroni test was performed to see the difference in total cholesterol averages within each group.

It was seen that there was a difference in average total cholesterol levels between K0 (107.5 ± 1.87 mg/dl) vs K1 (120.3 ± 5.53 mg/dl), ( *p* = 0.001). This shows that in the K1 group (positive control) given quail yolk succeeded in increasing cholesterol in mice with a significant difference compared to the K0 group (negative control), which was only given aquadest. Besides, differences in total cholesterol levels in the K1 group (120.3 ± 5.53 mg/dl) compared with K2 (107.3 ± 3.61 mg/dl), K3 (106.8 ± 4.57 mg/dl) and K4 (106.7 ± 0.51 mg/dl) groups showed that a significant decrease in total cholesterol levels ( *p* = 0.001). This proves that CE is efficacious in reducing total cholesterol levels, while in groups K2, K3, and K4, which were all given CE, there was not a significant difference between total cholesterol levels ( *p* > 0.05).

**Figure 2.**
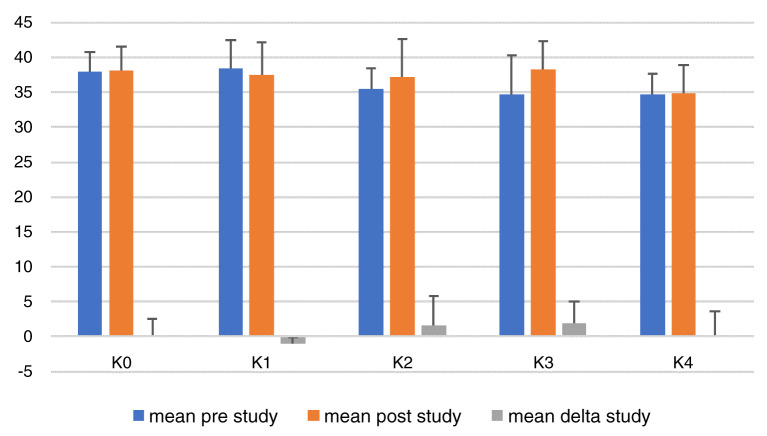
Total body weight per treatment group before (in day 14) and after (in day 28) treatment. K0, negative control group, no treatment or high-fat food (aquadest); K1, positive control group, diet of high-fat containing food (HFC; quail yolk); K2, HFC + cinnamon extract (CE) dose 2mg/20g body weight (BW); K3, HFC + CE dose 4mg/20gBW; K4, HFC + CE dose 8mg/20gBW. *Mean delta = (average BW at the end of the study) - (average BW at the beginning of the study).

Figure 2 shows that there were increases in BW in all groups. The largest increase of BW was in group K2, which was the positive control group who were given HFC quail yolk at 0.5 ml/20gBW. The smallest increase in BW was found in group K4, which was the group provided with HFC quail yolk and CE with the dose of 8 mg/20gBW (highest dose in this study). One-way ANOVA showed that there was no significant difference in BW between groups ( *p* = 0.419), which could be inferred that the action of giving CE gave no effect increasing BW in mice.

## Discussion

The aim of study to investigate cholesterol levels can be lowering in mice given variation of doses CE after consumption of quail yolk for a high-fat diet. Mice was used in this study because they have a similar biology to human, and can therefore be a model for human hyperlipidemia ^9– 11^. In this study, it was proven that consumption of 0.5ml quail yolk for 28 days increased the total cholesterol level between the negative and positive control groups ( *post-hoc* Bonferroni K0 vs K1; *p*=0.001).

There was a significant difference in the total decrease in cholesterol after treatment among all groups in this research. However, this is not consistent with Vanessa *et al*. (2013) who stated that there was a decrease, eventhough the difference is not statistically significant. Their studies were only done for 14 days, with an increase of 3x the initial dose (14.4 mg to 43.2 mg), while our study was carried out 28 days, leading to differing results ^15^.

Cholesterol is formed by the action of HMG-CoA reductase enzyme (3- *hydroxy*-3- *methylglutaryl*-CoA). If hypercholesterolemia is left without implementing proper diet or treatment, it can cause occlusion in a blood vessel. Hypercholesterolemia treated to medicines, such as statin could be given ^16^. The use of statins will competitively block HMG-CoA reductase and efficiently reduce serum LDL cholesterol. But, treatment using statin can cause rhabdomyolysis ^4^. Thus, it needed research on traditional or herbal medicine, e.g. CE, that needs developed because CE is predicted to have minimal adverse effects.

Cinnamon ( *Cinnamomum burmannii*) has cinnamaldehyde as it’s biggest compound. Cinnamaldehyde, a phenolic component abundantly found in *Cinnamomum* ^7^. Bandara *et al.* (2011) stated that cinnamon had the ability to be an antioxidant, antivirus, antifungal, antimicrobe, antitumor, and can lower cholesterol and blood pressure ^17^. Cinnamon is believed to have a direct role in lipid metabolism to prevent hypercholesterolemia and hypertriglyceridemia, as well as in preventing free fatty acids with its strong lipolytic activity ^18^. In the present study we believe that the increase of cholesterol levels due to quail yolk could be decreased with cinnamon maybe caused by its ability to block HMG-CoA reductase enzyme and suppress lipid peroxidation through increased antioxidant enzyme activity ^19^. Abeysekera *et al.* (2017) reported cinnamaldehyde that highest compound in bark extracts of Ceylon cinnamon possess moderate cholesterol esterase and cholesterol micellization inhibition and bile acid binding in vitro. It could lower cholesterol levels because it contributed to bile acid synthesis ^20^.

Our study showed that there was no difference in BW of the mice between groups ( *p* = 0.419). This is not consistent with Vafa *et al.* (2012) stated that consuming 3 grams of cinnamon for 8 weeks could decrease some biochemical and anthropometric variables compared to previous states significantly, i.e. a decrease of 1.19% body weight, 1.54% body mass index and 1.36% body fat. They conducted clinical study in T2DM patients who were given cinnamon supplements ( *Cinnamomum zeylanicum*). In addition to lowering BW variables, it could also decrease fasting blood glucose level by about 9.2%, and 6.12% of HbA1c and 15.38% of triglyceride levels ^21^. In contrary, Alsoodeeri *et al.* (2020) reported weight gain in rats (Albino rats) which are given Cinnamomum cassia after consuming high fat compared to body weight at the beginning until end of the study in each groups ^18^.

Leaf and Antonio (2017) stated that the higher the food intake (with Fat Mass/Fat Free Mass), the higher the increase in BW ^22^. In the present study, in addition to standard food intake, giving hypercholesterol and interventional food should increase BW, because the mice had increased energy intake. The amount of food intake will affect the amount of energy intake, which will be saved as fat and impact on the mice’s BWs, since energy intake is inversely proportional to physical activity. A high-fat diet group has low sensitivity to leptin, which results in increased appetite and food intake, thus increases the BW ^16^. However, this was not the case in our study.

## Conclusion

In the present study showed cinnamon ( *Cinnamomum burmannii*) extracts proved can be lowering of total cholesterol level for 14 days after consuming high-fat containing foods in mice models compared to does not given cinnamon.

## Data availability

### Underlying data

Figshare: data Analysis-cholesterol and bodyweight of mice.docx, https://doi.org/10.6084/m9.figshare.11901174.v2 ^23^

Data are available under the terms of the Creative Commons Attribution 4.0 International license (CC-BY 4.0).

## Acknowledgments

The author gives appreciation and thanks to the Pharmacology Laboratory of the Faculty of Medicine, Universitas Sumatera Utara for allowing the author to use the facility for collecting data in this research. In addition, thanks to all lecturers and examiners who had given advice and suggestions to the author, and all other parties who had contributed to this research.
