# Supplementary material for: The benefit of cinnamon ( Cinnamomum burmannii) in lowering total cholesterol levels after consumption of high-fat containing foods in white mice ( Mus musculus) models
Source: F1000Res. 2020 May 29;9:168. Originally published 2020 Mar 6. [Version 2] doi: 10.12688/f1000research.22311.2 (PMC7295078; doi:10.12688/f1000research.22311.2)
Supplement: Peer review history

# Peer review: Reviewer response for version 2

**DOI:** 10.5256/f1000research.26537.r64105

**Published:** 2020-06-12

**Authors:** Tahereh Farkhondeh

**Affiliations:** 1 Cardiovascular Diseases Research Center, Birjand University of Medical Sciences, Birjand, Iran

It is acceptable.

Is the work clearly and accurately presented and does it cite the current literature?

Partly

If applicable, is the statistical analysis and its interpretation appropriate?

Partly

Are all the source data underlying the results available to ensure full reproducibility?

Partly

Is the study design appropriate and is the work technically sound?

Partly

Are the conclusions drawn adequately supported by the results?

Partly

Are sufficient details of methods and analysis provided to allow replication by others?

Partly

Reviewer Expertise:

metabolic disorder

I confirm that I have read this submission and believe that I have an appropriate level of expertise to confirm that it is of an acceptable scientific standard.

# Peer review: Reviewer response for version 1

**DOI:** 10.5256/f1000research.24610.r61729

**Published:** 2020-04-14

**Authors:** Mourad Akdad

**Affiliations:** 1 Department of Biology, Faculty of Science and Technology Errachidia (FSTE), Errachidia, Morocco

This study aimed to evaluate the effect of cinnamon extract (C. E) on cholesterol levels in mice with hypercholesterolemia.  Authors demonstrated that C. E is useful to decrease cholesterol level, so its a promising plant to explore in the treatment of hypercholesterolemia and the related illness (hyperlipidemia, diabetes, atherosclerosis…).

1. Cinnamon has been shown to reduce total cholesterol, low-density lipoprotein and triglyceride levels, as well as increasing high-density lipoprotein levels. » precise the model of the study.
2. In introduction section, you must conclude by the aim of your research, this phrase “Mice were used in this study because of they have a similar biology to humans, and can therefore be a model for human hyperlipidemia8–10. must be include in discussion.
3. Avoid repetition such as: “This study was conducted at the Pharmacology Laboratory of the Faculty of Medicine, Universitas Sumatera Utara, Indonesia. » « The experiments took place at the Laboratory of Pharmacology Universitas Sumatera Utara (USU). »
4. What is the type of extract (aqueuous, ethanolic…..), and the part of plant by which industry do extraction.
5. Try to explain why you don’t have a dose-dependent effect in your experiment.
6. « Figure 1 shows that groups K0 (negative controls), K1 (positive controls), K2 (CE 2 mg/20gBW), K3 (CE 4 mg/20gBW), and K4 (CE 8 mg/20gBW), had a decrease in total cholesterol levels. » why all groups have a decrease of Total Cholesterol, you don’t precise the days of this results you must clarify the idea.
7. In the section results, days of measurement must be righted in the description to clarify the idea.
8. The smallest increase in BW was found in group K5 », K5 ??, you would like to say K4?
9. In the present study we believe that the increase of cholesterol levels due to quail egg yolk could be decreased with cinnamon by its ability to block HMG-CoA reductase enzyme and suppress lipid peroxidation through increased antioxidant enzyme activity18. » the mechanism involved in the antihypercholesterolemic effect must be declared with precaution, ‘may be’.
10. Our study showed that there was no difference in BW of the mice between groups (p = 0.419). This is not consistent with Vafa *et al.* (2012) ^1^ who stated that consuming 3 grams of cinnamon for 8 weeks could decrease some biochemical and anthropometric variables compared to previous states significantly, i.e. a decrease of 1.19% body weight, 1.54% body mass index and 1.36% body fat. In addition to lowering BW variables, it could also decrease fasting blood glucose level by about 9.2%, and 6.12% of HbA1c and 15.38% of triglyceride levels20. » model used by the study cited must be declared.
11. In the present study, a high-fat containing food used in combination with cinnamon extract ( *Cinnamomum burmannii*) for 28 days could decrease total cholesterol levels in mice. » this conclusion must be reformulated, high-fat containing food was used only to have the model for studying the effect of CE on cholesterol level, so the conclusion must be related only to CE.

Is the work clearly and accurately presented and does it cite the current literature?

Yes

If applicable, is the statistical analysis and its interpretation appropriate?

Yes

Are all the source data underlying the results available to ensure full reproducibility?

Yes

Is the study design appropriate and is the work technically sound?

Yes

Are the conclusions drawn adequately supported by the results?

Yes

Are sufficient details of methods and analysis provided to allow replication by others?

Yes

Reviewer Expertise:

Ethnopharmacology, medicinal plants, diabetes, hypertension

I confirm that I have read this submission and believe that I have an appropriate level of expertise to confirm that it is of an acceptable scientific standard.

## References

[1] : Effects of cinnamon consumption on glycemic status, lipid profile and body composition in type 2 diabetic patients. *Int J Prev Med*.2012;3(8) :531-6 22973482PMC3429799

# Peer review: Reviewer response for version 1

**DOI:** 10.5256/f1000research.24610.r61018

**Published:** 2020-04-02

**Authors:** Tahereh Farkhondeh

**Affiliations:** 1 Cardiovascular Diseases Research Center, Birjand University of Medical Sciences, Birjand, Iran

The present study investigated the effect of cinnamon extract on body weight and cholesterol levels of mice-fed HFD. It is a good idea.

 Some revisions should be done:

**Abstract**

1. In the method section several point should be cleared:
  1. Type of cinnamon extract.
  2. Female  or male mice.

**Introduction**

1. The aim of study should be better mentioned. it was not clear the question about the effect of plant on the lipid profile. In the past paragraph, the author described the lipid lowing effect of extract. Did this evidence extracted from traditional medicine reports?

**Method**

1. Did you used xylazine with ketamine for analgesic effect It should be mentioned. Animal ethics did not accept this anesthesia procedure. 
2. Please more about cholesterol measurement (technique, instrument, kit).

**Conclusion**

1. If you tested the quail egg yolks as a high-fat diet that induces hyperchloestromia. It is necessary in the abstract and introduction in the aim section.
2. In the first paragraph, you should about your findings without analytic indicator such as p-value.
3. You did not measure the changes in TG or FFA therefore, you should not concluded about in the conclusion section.

Is the work clearly and accurately presented and does it cite the current literature?

Partly

If applicable, is the statistical analysis and its interpretation appropriate?

Partly

Are all the source data underlying the results available to ensure full reproducibility?

Partly

Is the study design appropriate and is the work technically sound?

Partly

Are the conclusions drawn adequately supported by the results?

Partly

Are sufficient details of methods and analysis provided to allow replication by others?

Partly

Reviewer Expertise:

metabolic disorder

I confirm that I have read this submission and believe that I have an appropriate level of expertise to confirm that it is of an acceptable scientific standard, however I have significant reservations, as outlined above.

# Response

**Published:** 2020-04-06

**Authors:** yunita sari pane

**Affiliations:** universitas sumatera utara, Indonesia

RESPONSE to review's **Tahereh Farkhondeh**

Cardiovascular Diseases Research Center, Birjand University of Medical Sciences, Birjand, Iran

The present study investigated the effect of cinnamon extract on body weight and cholesterol levels of mice-fed HFD. It is a good idea.

Some revisions should be done:

**Abstract**

1.In the method section several point should be cleared:

    1.Type of cinnamon extract.

**Answer:**

Type of cinnamon extract used in this study had been explained part of METHODS (page 3 manuscript in F1000research).

We used Cinnamomum burmannii in powder extract.

     2. Female or male mice.

**Answer:**

The sample animal were male mice. This is one of the inclusion criteria of this study. Its mean to homogenized (make smaller of the differences in each samples) for prevent BIAS. As we known, there are differences between male and female will increasing number of lipid and dysfunction metabolic, including of hyperlipidemia. It related to level of hormones.  

**Introduction**

1. The aim of study should be better mentioned.

**Answer:**

Thank you for your comment, we would confirm our statement on the aim of this study.

The aim of the study to determine cinnamomum burmannii effective in reducing total cholesterol levels of mice (Mus musculus) given high fat feed.

It was not clear the question about the effect of plant on the lipid profile.

**Answer:**

We had explained about the effect of plant on the lipid profile related to few references.

Please see in part of DISCUSSION (page 5 manuscript in F1000reseach).

Cinnamon is believed to have a direct role in lipid metabolism and preventing hypercholesterolemia and hypertriglyceridemia, as well as in preventing free fatty acids

with its strong lipolytic activity17.

In the present study we believe that the increase of cholesterol levels due to quail egg yolk could be decreased with cinnamon by its ability to block HMG-CoA reductase enzyme and suppress lipid peroxidation through increased antioxidant enzyme activity18.

In research by Pai *et al*. (2013), it was stated that cinnamaldehyde could lower cholesterol and triglyceride levels by the action of some enzymes secreted in certain amounts, which might contribute to bile acid synthesis19.

(This study needed continuos some research in the future to measured number of cinnamaldehyde in Cinnamon Extract Powder ^® ^ per 1 mg CE used. Then we’ll do new research to prove how CE mechanism as anti hypercholesterolemia, Is it blocked by HMG-CoA reductase enzyme and suppress lipid peroxidation through increased antioxidant enzyme activity or dysfunction of bile acid synthesis or both mechanism) ?

In the past paragraph, the author described the lipid lowing effect of extract. Did this evidence extracted from traditional medicine reports?

**Answer:**

Yes, the explain about “this has been suggested as a substance……triglyceride levels”  better

moves to part of DISCUSSION, because we took from one of reference. We will add inform about the quail egg yolks as a high-fat diet that induces hypercholesterolemia in part of ABSTRACT.

We can not add inform in the AIM of study. Because, if we added in the Aims of study, it was not consequence to the title of study. If we changed title "Benefit of cinnamon (Cinnamomum burmannii) in lowering total cholesterol level after consumption of  the quail egg yolk as a high-fat diet that induces hypercholesterolemia". It will make the title very long and not in accordance to the rules of journals F1000Research.

**Method**

1. Did you used xylazine with ketamine for analgesic effect It should be mentioned. Animal ethics did not accept this anesthesia procedure.

**Answer:**

Yes, we did. We corrected uncomplete in methods ketamin with xylazine. We choosed of **Injectable Anesthetic Agents, we used **Ketamine/xylazine* We follow to GUIDELINES - PREPARATION OF KETAMINE/XYLAZINE COCKTAIL FOR MICE. Dose Ketamine 80-100 mg/kg intra peritoneal (IP) and Xylazine 10-12.5 mg/kg IP.(reference 11. Vertebrate Animal Research. The University of Iowa).

The administration of effective anaesthetic agents to avoid impact/effects of the experimental procedures when we did cut off mice tail to collect blood sample  with anesthesia to make minimize pain and prevent distress caused of pain in mice.

 

1. Please more about cholesterol measurement (technique, instrument, kit).

**            **


**ANSWER:**

In Laboratory analysis study showed how was technique to take blood from tail

*correction in methods study cut of mice tail.

“Mice tails were cut 2 – 5 mm to draw blood, which was then assessed for total cholesterol using the digital autocheck ^®^(merck of digital cholesterol measured tool). We used stick cholesterol to measured total cholesterol of mice.

Regarded to follow **National Centre  for the Replacement, Refinement & Reduction (NC3Rs) of Animals in Research **to measured (page 4 manuscript in F1000reseach).

**Conclusion**

1. If you tested the quail egg yolks as a high-fat diet that induces hyperchloestromia. It is necessary in the abstract and introduction in the aim section.

**Answer:**

Yes you right. We had tried to add in abstract before, but it was more than 250 words, it was     

 not allow with Journal F1000research rules. But we will try again to put in your comment in   

 abstract.

 

1. In the first paragraph, you should about your findings without analytic indicator such as p-value.

**  Answer:**

  Im sorry, I can’t catch what you mean in this statement. Would you like to describe more detail?

 

1. You did not measure the changes in TG or FFA therefore, you should not concluded about in the conclusion section.

**Answer:**

Yes, thank you for your comment. We are agree with you.

Conclusion:

The cinnamon extract (Cinnamomum burmannii) proved it can be decrease of total cholesterol levels of for 14 days in mice consuming high-fat containing compared to group without given CE.
